# Within-Session Matching Analyses to Quantify the Effects of Individualized Preferred Interactions on Social Time Allocation

**DOI:** 10.3390/bs16071169

**Published:** 2026-07-11

**Authors:** Pierce M. Taylor, Celeste M. Tevis, Samuel L. Morris

**Affiliations:** Department of Psychology, Louisiana State University, 236 Audubon Hall, Baton Rouge, LA 70803, USA; ptayl33@lsu.edu (P.M.T.); celeste.tevis@childrenscolorado.org (C.M.T.)

**Keywords:** matching law, preference assessment, sociability, social reinforcement, quantitative analysis

## Abstract

Researchers have used the generalized matching equation to quantify the function of social interaction based on within- and across-session patterns of social time allocation. Within-session matching analyses could provide an efficient method of quantifying the extent to which preferred, individualized social interactions are more reinforcing than generic, non-individualized social interactions. The current study explores the potential utility of this novel application of the generalized matching equation. We recruited four participants who displayed indifference during baseline sociability assessment sessions. Individualized, preferred interactions were identified for each participant and then introduced during subsequent assessment sessions. Within-session matching analyses of the last session of baseline and first session of intervention suggested improvements in sensitivity to social reinforcement and reductions in bias toward a given side of the assessment for three out of four participants. Results were compared to a reanalysis of previously published data for three participants experiencing an identical intervention. Heterogeneity in the persistence of intervention effects as well as implications for future research related to increasing social time allocation and applying the generalized matching equation are discussed.

## 1. Introduction

Choice refers to the allocation of behavior across available response alternatives. In basic behavior analytic research, choice is often studied using concurrent operant arrangements in which two or more response alternatives produce reinforcement according to different contingencies. For example, the relative rate of reinforcement provided by the two alternatives may systematically vary across conditions. The generalized matching equation suggests that the relative rate of responding across two alternatives tends to approximately match the relative rate of reinforcement produced by those alternatives:

$$\log\left(\frac{B_{1}}{B_{2}}\right)=alog\left(\frac{R_{1}}{R_{2}}\right)+logb$$

where $B_{1}$ and $B_{2}$ denote response rates for the two alternatives, $R_{1}$ and $R_{2}$ denote the corresponding rates of reinforcement produced by each alternative. The equation includes two free parameters, *a* and *b*, that adjust the slope and y-intercept of the relation, respectively, to best describe the behavior of each individual subject. The *a* parameter is interpreted as a measure of sensitivity to differences in relative reinforcement rate, whereas *b* is interpreted as a measure of bias toward a particular response alternative due to variables other than relative reinforcement rate. An extensive body of experimental work with non-human animals suggests that the generalized matching equation provides a high-quality description of choice in concurrent operant arrangements (i.e., *a* ≈ 0.8, *R*^2^ ≥ 0.9; Baum, 1979; Davison & McCarthy, 2016; McDowell, 2013; Wearden & Burgess, 1982). Ultimately, this literature suggests that non-human animals’ choice is determined, at least in part, by the relative rate of reinforcement they have experienced for the available alternatives.

Researchers have also investigated whether human choice may be well described by the generalized matching equation and accounted for by relative reinforcement rate. Initial experimental research with human participants yielded more variable results than those observed with non-human animals, failing to consistently produce data that were well described by the generalized matching equation (Pierce & Epling, 1983; Schmitt, 1974; Schroeder, 1975; Wurster & Griffiths, 1979). However, subsequent work has shown that additional procedural variables, such as the addition of ordinal discriminative stimuli (Bradshaw et al., 1976) and inclusion of denser schedules of reinforcement (Klapes et al., 2020, 2021), can produce human behavior that is more sensitive to relative reinforcement rate and well aligned with the predictions of the generalized matching equation. Although this remains an ongoing area of inquiry, research to date suggests that human choice is also determined, at least in part, by the relative rate of reinforcement.

It may also be useful to apply the generalized matching equation to investigate socially significant human behavior. Matching analyses may yield more precise quantitative outcomes than standard assessment approaches, generate testable predictions about choice, and connect applied findings to extensive basic literature. These characteristics position matching analyses as a mechanism for improving assessment methods for understanding the causes of behavior and intervention procedures for facilitating behavior change. Accordingly, researchers have extended matching analyses to a range of socially significant human behavior outside of laboratory preparations. Applications have included analyses of academic performance (e.g., Mace & Neef, 1994), conversational exchanges (Beardsley & McDowell, 1992; J. C. Borrero et al., 2007; Conger & Killeen, 1974; McDowell & Caron, 2010), sports performance (e.g., Falligant et al., 2021; Reed et al., 2006; Rotta et al., 2021; Vollmer & Bourret, 2000), and games (e.g., Cero & Falligant, 2020; Schenk & Reed, 2020). One area of significant clinical importance is the assessment and treatment of challenging behavior. Researchers have demonstrated that the generalized matching equation provides a framework for quantifying the relation between relative reinforcement rate and the relative occurrence of challenging behavior versus appropriate alternatives (C. S. W. Borrero et al., 2010; J. C. Borrero & Vollmer, 2002; Kronfli et al., 2021; McDowell, 1982, 1988; St. Peter et al., 2005). For example, C. S. W. Borrero et al. (2010) demonstrated that the relative rate of challenging versus appropriate behavior matched the relative rate of reinforcement produced by each type of behavior. Importantly, rather than categorical outcomes about the relative occurrence of appropriate and challenging behavior, matching analyses quantified the degree of sensitivity to relative reinforcement rate (i.e., higher *a* indicates a greater degree of change in behavior given a shift in relative reinforcement rate), bias toward challenging behavior (i.e., higher log *b* indicates more probable challenging behavior, regardless of variation in relative reinforcement rate), and the degree to which variation in behavior can be accounted for by relative reinforcement rate (i.e., higher *R*^2^ indicates that less variability in behavior is attributable to other variables).

Given that matching analyses have proven useful in quantitatively characterizing clinically meaningful behavior, it is possible that the generalized matching equation could also characterize the effects of interventions designed to alter relative response rate by quantifying changes in sensitivity and bias. Utilizing matching analyses to evaluate intervention effects may not only quantify changes in behavior but also elucidate whether and how the intervention alters the relation between choice and relative reinforcement rate. To our knowledge, Kronfli et al. (2021) is the only published study using matching analyses to evaluate intervention effects. They evaluated how children’s relative rates of appropriate and challenging behavior covaried with parents’ relative rates of reinforcement for each response type. During baseline observations, parents provided more reinforcement for challenging behavior, and their children displayed more challenging behavior, as predicted by the generalized matching equation. Next, parents received behavioral skills training related to differential reinforcement of appropriate behavior. During post-training observations, parents were more likely to provide reinforcement for appropriate behavior, and their children were more likely to display appropriate behavior. Importantly, the degree of improvement in child behavior was commensurate with the degree to which parents differentially reinforced appropriate behavior, as predicted by the generalized matching equation. Kronfli et al. demonstrated that matching analyses can help explain why and quantify how behavior changes following intervention. However, their procedures did not produce data suitable for fitting the generalized matching equation to individual participant’s behavior. As a result, estimates of sensitivity and bias could not be obtained for each participant and aggregation across participants may have obscured important individual differences. Researchers have yet to apply the matching analyses to evaluate within-participant changes in sensitivity and bias in response to intervention. Doing so may allow for more quantitative conclusions about intervention efficacy, uncover meaningful heterogeneity across individual participants, and elucidate possible mechanisms of behavior change. For example, across different individuals, an intervention may change behavior by increasing sensitivity to relative reinforcement rate (quantified by *a*), shifting bias toward a desired alternative (quantified by *b*), diminishing the influence of variables other than relative reinforcement rate (quantified by *R*^2^), or some combination of these mechanisms.

Another area of clinical research to which researchers have applied the generalized matching equation is assessing the social time allocation of children with autism (Call et al., 2013; McKeown et al., 2026; Morris & Vollmer, 2020b, 2021, 2022a). Sociability assessments are a naturalistic, play-based assessment that yield a general measure of the function of social interaction based on participant’s time allocation. During sociability assessments, a room is divided into two sides, each equipped with chairs, a table, and developmentally appropriate toys. A therapist with whom the participant is unfamiliar sits on one side of the room. This side is designated the social side, because when the participant is also on that side of the room the therapist provides generic, non-individualized interactions such as descriptions of the participant’s behavior or environment and praise-like statements (e.g., “Playing with you is fun!”). More individualized interactions are delivered only contingent on clear mands (i.e., vocal, gestural, or other communicative responses that specify a particular type of play or interaction; Skinner, 1957). When the participant is on the side of the room without therapist (i.e., the independent play side), toys remain available, but no social interactions are provided. Within each session, the therapist periodically switches sides of the room to alter the availability of social interactions and observe the effects on participant behavior.

The results of sociability assessments may be useful in at least a couple of ways. First, like skills assessments (e.g., Partington, 2008; Sundberg, 2014) or assessments of challenging behavior (e.g., Allen et al., 2026; Deshais et al., 2024), sociability assessments can be conducted at the onset of and intermittently throughout behavior analytic services to monitor changes in the function of social interaction and fundamental social behavior. Second, sociability assessments may be used to identify participants who display indifference to or avoidance of social interaction and may benefit from intervention designed to improve the function of social interaction. For example, sociability assessment sessions indicating social avoidance may provide a useful baseline with which to evaluate the effects of individualized, preferred interactions (Morris & Vollmer, 2022b), conditioning procedures to improve the function of social interaction (e.g., Axe & Laprime, 2017; Jimenez-Gomez et al., 2024; P. M. Taylor et al., 2025), or establish social play or communication skills (e.g., Agana et al., 2025; Mattson et al., 2023; Seaver & Bourret, 2020; B. A. Taylor & Hoch, 2008).

Traditionally, sociability assessments have yielded categorical outcomes regarding the function of social interaction. Participants are characterized as social, indifferent, or avoidant based on whether they allocate greater than 55%, 45–55%, or less than 45% of their time on the social side, respectively. Morris and Vollmer (2022a) modified the sociability assessment procedures to titrate the relative amount of time that social interaction was available on each side of the assessment across several conditions. This adapted procedure produced data suitable for fitting the generalized matching equation. The *a* values produced by this analysis precisely quantified the extent to which participant’s time allocation was sensitive to social reinforcement based on the degree to which time allocation changed based on variation in the relative availability of social interaction. Additionally, *R*^2^ values indicated that most of the variation in participants’ behavior could be accounted for by the relative availability of social interaction, suggesting that results were well described by an adapted generalized matching equation. More recently, Morris and Pizzuto (2023) extended this approach by capitalizing on within-session variation in the availability of social interaction to produce data suitable for fitting the generalized matching equation to the results from a single sociability assessment session. As in Morris and Vollmer’s extended analysis, these within-session analyses quantified sensitivity to social reinforcement and captured greater variation in sensitivity across participants than was evident based on across-session measures of the duration on the social side.

Researchers have yet to apply these analyses to quantify the effects of interventions designed to improve sociability and increase social time allocation. Morris and Vollmer (2022b) demonstrated that providing individualized, preferred social interactions could increase social time allocation for three children who displayed indifference to or avoidance of generic social interactions. However, researchers have yet to replicate these procedures. Utilizing the matching analyses described by Morris and Pizzuto (2023) to evaluate the effects of individualized, preferred interactions could extend the literature on sociability assessments and applications of the generalized matching equation. Doing so would quantify the extent to which individualized, preferred interactions (a) increase *a* or sensitivity to social reinforcement, (b) alter *b* or bias toward a given side of the arrangement, and (c) increase *R*^2^ or the degree to which social time allocation systematically varies with social reinforcement on a within-participant basis. Therefore, purpose of this study was to apply within-session matching analyses to quantify the effects of individualized, preferred interactions on the social time allocation of children with developmental disabilities. Specifically, the current study systematically replicates the intervention evaluated by Morris and Vollmer (2022b) and replicates and extends the matching-based analysis proposed by Morris and Pizzuto (2023) by applying it to quantify intervention effects on a within-participant basis.

## 2. Method

This manuscript begins with novel analyses of the data from the three participants (Felicity, Edward, and Lola) in Morris and Vollmer (2022b). Then, we include data from four additional participants (Arthur, Axel, Candace, and Travis) who were recruited for the purpose of this study and whose data have not been previously published. The procedures used with both groups of participants were identical and are described in full below. The procedures were approved by Louisiana State University’s Institutional Review Board.

### 2.1. Participants and Setting

All participants were recruited from a local clinic providing behavior analytic services and were diagnosed with a neurodevelopment disorder; six participants were diagnosed with autism spectrum disorder, and one participant with a global developmental delay. Participants were recruited based on clinical concerns related to the function of social interaction and the efficacy of differential social consequences in facilitating skill acquisition. The participants’ age, race and ethnicity, sex, and primary form of communication are listed in Table 1. All participant names are pseudonyms. Caregivers provided written informed consent for their child’s participation. Consent was obtained by the last author before the participant’s involvement in the study. Sessions were conducted in therapy rooms located within the clinic from which participants were recruited. Sessions for Edward, Felicity, and Lola were conducted in the same 3.1 m by 3.4 m room, whereas sessions for Arthur, Axel, Candace, and Travis were conducted in the same 3.4 m by 2.6 m room. The room was divided in half with a line of white tape, and each side included a table, a set of chairs, and a variety of developmentally appropriate toys (e.g., blocks, cars, and puzzles). Sessions were conducted by the first and second authors, and research assistants collected data from the live video feed or video recordings.

### 2.2. Experimental Design

The current study employed two types of single case experimental designs at two separate levels of analysis. First, at a molar level, we evaluated the effects of individualized, preferred social interactions on social time allocation using a nonconcurrent multiple baseline design in which the number of baseline sessions that were conducted prior to the introduction of the intervention varied across participants. This arrangement controlled for familiarity or repeated exposure as an alternative explanation for increases in social time allocation. Second, at a more molecular level, we capitalized on the within-session reversal design incorporated within each sociability assessment session. As described by Morris and Pizzuto (2023), the side of the room on which social interactions were available alternates every 2 min during each assessment session, which approximates a within-session reversal design that could allow a single session to yield clear conclusions about how social interaction influenced participants’ time allocation. Therefore, the only way the participant could consistently maintain access to or avoid social interaction is by changing their behavior based on changes in therapist location and the availability of social interaction. Incorporating both across and within-session experimental designs may improve precision or clarity and may be useful because the two designs may allow for distinct, yet complementary, conclusions.

### 2.3. Procedure

#### 2.3.1. Baseline

The baseline condition followed the procedure outlined by Morris and Vollmer (2021). Sessions were conducted by a novel therapist with whom the participant had no prior learning history. At the beginning of each session, the participant entered the room, and a research assistant would instruct them to, “Go play wherever you want.” The therapist started the session on one side of the divided room, and the side where the therapist began alternated across sessions. Sessions were 8 min in duration. The therapist switched sides every 2 min (i.e., fixed-time 2-min schedule) and signaled a change in the availability of social interaction by saying something like, “I’m going to go play over here.”

The side of the room on which the therapist was present was designated the social side because the therapist would provide generic social interactions at least once every 10 s when the participant was on the social side, similar to a variable-interval 10-s schedule. Social interactions included vocal descriptions of the participant’s behavior and the environment (e.g., “Look at you, you’re dancing.”), praise statements (e.g., “Wow, you’ve got some great moves!”), and participant-directed play (e.g., helping the participant build a block tower after they begin stacking blocks independently). The therapist also reinforced clear mands for individualized forms of play or social interaction (e.g., tossing a ball after the participant vocally manded for the therapist to throw the ball or handed the therapist the ball). The opposite side of the room where the therapist was not present was designated the independent play side^1^, because no social interactions were available on that side of the room. When the participant was on the independent play side, the therapist maintained a neutral gaze and body orientation but did not interact with the participant. One exception to this general rule was that the therapist reinforced mands for therapist movement. For example, if the participant asked the therapist to come play or hand-led them over to the other side, the therapist walked to the side of the room where the participant was located.

#### 2.3.2. Graphics Interchange Format Paired Stimulus Preference Assessment

After completing the baseline condition, a Graphics Interchange Format (GIF) paired-stimulus preference assessment (PSPA) for social interaction was conducted (Morris & Vollmer, 2020a). The purpose of this assessment was to identify individualized, preferred social interactions to be utilized during the subsequent intervention. The assessment included eight possible options: seven forms of social interactions and one control option (i.e., no programmed consequences)^2^. Informal interviews with two or more clinicians who regularly worked with the participant were used to determine social interactions. Interactions reported by both clinicians were included. If additional social interactions were needed, those reported by only one clinician or those to which the participant responded positively during informal probes were included. The assessment was conducted by a research assistant familiar with the participant, not the therapist who completed the baseline, exposure, or intervention sessions. This arrangement was used to limit the influence of the preference assessment on the participant’s time allocation with the therapist. The GIFs included in the assessment displayed the participant and research assistant engaging in each interaction for 5–10 s.

Prior to the start of the PSPA proper, exposure trials were conducted so the participant contacted the contingency for selecting each GIF, with the aim of facilitating discrimination between the available options. During exposure trials, the research assistant presented the tablet with one GIF, and the participant was prompted to select the GIF. Contingent on a selection, the research assistant provided the corresponding type of social interaction for about 10 s. During the exposure and the assessment proper, selection of the control option resulted in the therapist orienting away from and not interacting with the participant for 10 s. A random number generator predetermined the order of assessment and exposure trials. After the exposure, GIFs corresponding to each type of social interaction were presented with each other GIF in a pair-wise manner until all response options had been presented together once.

At the start of each trial, the research assistant presented a screen depicting two GIFs to the participant and instructed them to, “Pick one.” If the participant did not select within 5 s of the trial presentation, the research assistant removed and represented the tablet. No selection was recorded if the participant did not select a consequence within 5 s after representing the trial. The percentage of trials in which each stimulus was selected, calculated based on the number of trials in which the stimulus was presented, was used to create a preference hierarchy. The three most selected consequences were included in the exposure and intervention sessions. If the control was one of the three most-selected consequences, the fourth most selected consequence was also used in the intervention. Arthur’s most preferred interactions were zaps (i.e., fast, repetitive shakes while saying “Zap!”), tossing a ball back and forth, and push-and-pull (i.e., holding hands while the therapist pushed and pulled). Axel’s top selections were tickles, the control, dancing, and dips (i.e., sitting on the therapist’s lap and leaning back and forth). Candace’s most preferred interactions were rocket ship (i.e., being lifted up by the therapist), tickles, and zaps. Travis’ most preferred interactions were nose boops (i.e., lightly tapping his nose and saying “boop”), spinning, and zaps.

#### 2.3.3. Exposure Session

After the completion of the preference assessment and before the start of the intervention, an exposure session was conducted to ensure that participants contacted the change in type of social interaction available. The exposure consisted of a 5-min abbreviated sociability assessment in which the therapist switched sides every 60 s. Participants were prompted to make social switches if they did not follow the therapist within 30 s of the therapist movement. When the participant was on the social side, the therapist initiated one of the three individualized, preferred social interactions about every 10 s (i.e., comparable to a VI 10 s schedule). Procedures for initiating social interactions are described below.

#### 2.3.4. Intervention

Intervention session procedures were identical to the baseline session procedures described above, with one exception: the addition of therapist-initiated individualized, preferred interactions. The therapists initiated social interactions by extending their arms toward the participant and saying something similar to, “Do you want (name of interaction)?” If the participant approached the therapist or did not display signs of disinterest, the social interaction was delivered. If the participant displayed (i.e., moving or pushing away) or communicated (i.e., “No”, shaking head) disinterest, then the therapist did not deliver the social interaction. Contingent on the participant remaining on the social side for 3 s, the therapist initiated one of the three most highly preferred interactions indicated by the preference assessment. If the participant remained on the social side, the therapist initiated a highly preferred interaction every 30 s (i.e., similar to a variable interval 30-s schedule). The therapist honored mands for additional social interactions if the participant vocally manded for the interaction or directed the therapist via gestures or hand leading. As in the preference assessment, all individualized, preferred interactions took 5–10 s to complete.

### 2.4. Response Measurement

All sessions were video recorded from a camera that was mounted in the middle of the room at the top of the wall. The camera provided a live video feed on a connected device outside of the room. Research assistants collected data using a mobile data collection application, Countee, from the live video feed or video recordings of the session. The primary dependent variable was the duration on the social side. Participants were recorded as being on the social side when both feet were on the same side of the room as the therapist. If participants had one foot on both sides of the room or were standing on the line, they were recorded as being on the side they were previously on until both feet crossed the line. The percentage of time on the social side was calculated by dividing the total duration the participant was on the social side by the session duration and multiplied by 100. Social switches were recorded when the participant moved from the independent play to the social side or when the participant made a request for the therapist to come play on the same side as the participant. An avoidant switch was recorded when the participant moved from the social side of the room to the independent play side or emitted a mand to terminate social interaction. Therapist location on the left or right side of the room and movement between sides were recorded according to the location of their feet using the same criteria as duration on the social side.

#### Interobserver Agreement

A secondary observer collected interobserver agreement data for at least 20% of all baseline and intervention sessions for each participant. Interobserver agreement data were calculated using the proportional method by dividing the session into 10-s intervals and comparing each observer’s data interval-by-interval. Then, for each interval, the smaller duration was divided by the larger duration recorded. These quotients were then summed, divided by the total number of intervals, and multiplied by 100. Because the proportional quotient is undefined when both observers record zero occurrences or duration, those intervals were assigned an agreement value of 1 to represent agreement. The mean agreement score for Arthur, Axel, Candace, and Travis was 94.32% and ranged from 85.9–99.54% for duration on social side, 96.55% and ranged from 90.63–100% for social switches, and 95.72% and ranged from 85.42–100% for avoidant switches. Interobserver agreement scores for Edward, Felicity, and Lola are provided in Morris and Vollmer (2022b).

### 2.5. Data Analysis

#### 2.5.1. Within Session Visual Analysis

Within session visual analysis of response patterns were completed for the last baseline session and the first intervention session for each participant. This analysis allows us to graphically display the participant’s location relative to the therapist (i.e., where social interaction was available) within each session and may reveal molecular response patterns that are difficult to detect using across-session analyses. The session was divided into 10-s intervals, and the duration data were used to determine the percentage of seconds within each interval that the participant spent on the social side. Then, we used the therapist’s initial location and the time stamp of the therapist’s movement data to determine the therapist’s location within each 10-s interval. To facilitate comparison across sessions and participants, the data were normalized to display the percentage of each interval that the participant spent on the side on which the therapist began the session (referred to as side x, as in Morris & Pizzuto, 2023). In these analyses, 100% represents one side of the assessment room (e.g., the right side), and 0% represents the other (e.g., the left side). The percentage of each interval that the therapist and participant spent on side x was displayed across intervals to facilitate visual analysis.

#### 2.5.2. Matching Analysis

As described in Morris and Pizzuto (2023), five 90-s bins that captured within-session variance in the therapist location and the availability of social interaction were extracted from each session. The first bin, from Second 0 to Second 90, in which the therapist was on side x (i.e., the side where they began the session) for 100% of the time; a second bin, from Second 290 to Second 380, in which the therapist was on side x for 78% of the time; a third bin, from Second 200 to Second 290, in which the therapist was on side x for 56% of the time; a fourth bin, from Second 90 to Second 180, in which the therapist was on side x for 33% of the time; and a fifth bin, from Second 390 to Second 480, in which the therapist was on the side x 0% of the time. For each 90-s bin, we calculated the ratio of time the participant (i.e., P) and the therapist (i.e., T) spent on side x to time spent on the other side of the assessment (i.e., side y). Because we could not calculate a ratio for a given 90-s bin if the numerator or denominator was 0 s, we added 1 s to the bins with 0 s to ensure that ratios of the therapist and participant social time allocation were calculable. For each participant’s final baseline session and first intervention session, these values were input into the equation below, which is an adaptation of the generalized matching equation (Baum, 1974) from Morris and Pizzuto (2023):

$$\log\left(\frac{P_{x}}{P_{y}}\right)\;=\;a\;log\left(\frac{T_{x}}{T_{y}}\right)+\log b$$

#### 2.5.3. Adapted Risk Ratios

Adapted risk ratios (Joslyn & Morris, 2024) were calculated for each participant on a phase-by-phase basis to quantify changes in likelihood of social or avoidant switches given a therapist movement as a function of individualized, preferred interaction. Risk ratios were defined as the conditional probability of social or avoidant switches within 10 s of a therapist movement divided by the corresponding unconditional probability of a social or avoidant switch across all 10 s intervals. Risk ratios greater than 1.0 indicate an increased probability of switching following therapist movement relative to the unconditional probability of switching and values less than 1.0 indicate a decreased likelihood of switching following therapist movement relative to the unconditional probability of switching.

## 3. Results

### 3.1. Within-Session Visual Analysis

**Figure 1:**
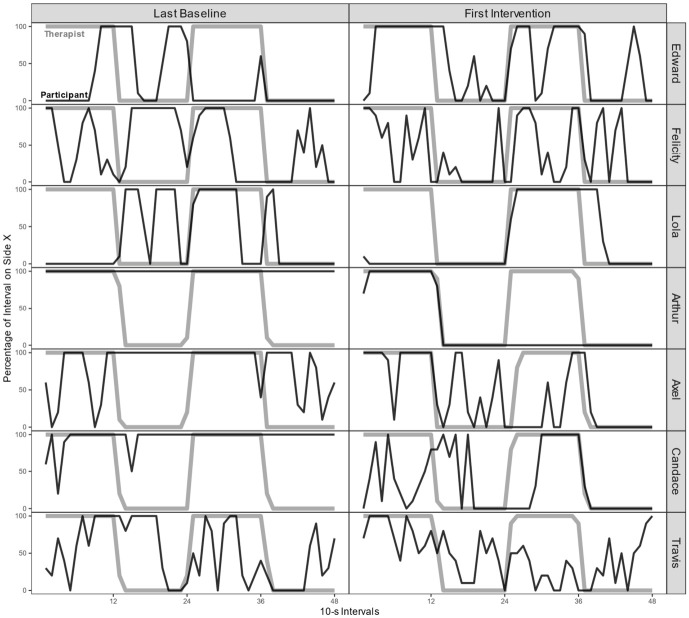
Within-Session Time Allocation for Therapist and Participant for the Last Baseline and First Intervention Sessions.

Each panel of Figure 1 displays the location of the therapist and participant during the last baseline and first intervention sessions. The y-axis displays the percentage of each interval the therapist and participant were on side x (i.e., the side on which the therapist started). The thicker grey line displays the therapist location, whereas the black line displays the participant location. The top row of panels displays Edward’s data. The within-session analysis of his last baseline session illustrates that Edward was indifferent to the availability of social interaction. Edward displayed one clear instance of social avoidance (i.e., movement away from the side of the room on which social interaction was available) following the second therapist movement at interval 24 during this session. During the first intervention session, Edward’s time allocation more closely corresponded with the therapist’s location, with clear, immediate social approach (i.e., movement to the side of the room on which social interaction was available) occurring following the second and third therapist movement. The second row of panels displays Felicity’s data. The within-session analysis of her last baseline session illustrates that Felicity’s time allocation across sides was variable. She displayed frequent switches and her time allocation did not vary systematically based on the therapist’s location. Felicity displayed social avoidance following the first therapist movement within this session. During the first intervention session, her time allocation across sides showed greater correspondence with the therapist’s location. Although she continued to switch sides frequently, she displayed social approach following the second and third therapist movement. The third row of panels displays Lola’s data. The within-session analysis of her last baseline session illustrates that Lola was largely indifferent to the availability of social interaction. Lola displayed two instances of social avoidance (i.e., first and third therapist movement), but also one instance of social approach (i.e., second therapist movement). During the first intervention session, Lola’s time allocation across sides more closely corresponded with the therapist’s location, with fewer instances of switching and more extended periods of time on the same side as the therapist. Lola displayed one clear, immediate instance of social approach during this session, following the second therapist movement.

The fourth row of panels displays Arthur’s data. The within-session analysis for Arthur’s final baseline session illustrates that he was indifferent to the availability of social interaction, as he remained on one side of the room throughout the session and never switched sides. During the first intervention session, Arthur displayed one clear, immediate instance of social approach following the first therapist movement but remained on that side of the room for the remainder of the session. The fifth row of panels displays Axel’s data. The within-session analysis of his last baseline session illustrates that Axel was indifferent to the availability of social interaction, as he remained on one side of the room for most of the session and displayed limited switching between sides. During the first intervention session, Axel’s time allocation across sides showed periods of correspondence with the therapist’s location, including two clear instances of social approach following the first and third therapist movement. The sixth row of panels displays Candace’s data. The within-session analysis of her last baseline session illustrates that Candace was indifferent to the availability of social interaction, as she largely stayed on one side of the room. During the first intervention session, her time allocation showed a moderate increase in correspondence with the therapist’s location, with more switches observed relative to baseline and one clear instance of social approach following the third therapist movement. The seventh row of panels displays Travis’ data. The within-session analysis of his last baseline session illustrates that Travis’ time allocation was variable and not systematically related to therapist location, and a similar pattern was observed during the first intervention session, indicating no change in sensitivity to the availability of social interaction.

Overall, within-session visual analysis suggests that most participant’s time allocation corresponded more closely to the therapist location and the availability of social interaction during the first session of intervention relative to the last session of baseline (Edward, Felicity, Lola, Arthur, Axel, Candace). Only Travis’ data suggest no change in the pattern of time allocation following the onset of intervention.

### 3.2. Matching Analysis

**Figure 2:**
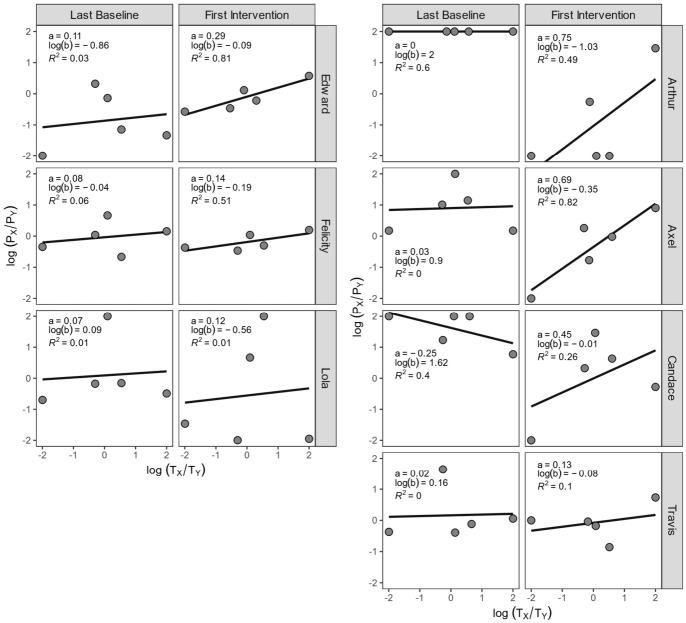
Within Session Matching Analyses for Last Baseline and First Intervention Sessions.

Figure 2 displays results of the within session matching analysis for each participant during the final baseline (left column) and first intervention session (right column). Each data point represents the therapist’s and participant’s relative time allocation across both sides of the room within one of the five 90-s bins described in the data analysis section. Edward’s data are displayed in the top row of the left group of graphs. Analysis of Edward’s last baseline session quantifies the degree to which he allocated his time relatively indifferently (i.e., *a* = 0.11) and displayed bias toward the side on which the therapist did not begin the session (i.e., *b* = −0.86). Edward’s time allocation did not systematically vary with therapist location and the availability of social interaction (i.e., *R*^2^ = 0.03). During the first intervention session, Edward demonstrated an increased sensitivity toward the availability of social interaction (i.e., *a* = 0.29) and less bias toward the side on which the therapist did not begin the session (i.e., *b* = −0.09). His time allocation varied far more systematically with therapist location and the availability of social interaction (i.e., *R*^2^ = 0.81).

Felicity’s data are displayed in the second row of the left group of graphs. During her last baseline session, she allocated her time indifferently (i.e., *a* = 0.08) and displayed a slight bias toward the side on which the therapist did not begin the session (i.e., *b* = −0.04). Felicity’s time allocation did not systematically vary with the availability of social interaction (i.e., *R*^2^ = 0.06). During the first intervention session, Felicity demonstrated a small increase in sensitivity toward the availability of social interaction (i.e., *a* = 0.14) and her time allocation varied more systematically with the availability of social interaction (i.e., *R*^2^ = 0.51). However, she also displayed slightly more bias toward the side on which the therapist did not begin the session (i.e., *b* = −0.19).

Lola’s data are displayed in the bottom row of the left group of graphs. During her last baseline session, she allocated her time indifferently (i.e., *a* = 0.07) and displayed a slight bias toward the side on which the therapist began the session (i.e., *b* = 0.09). Lola’s time allocation did not systematically vary with the availability of social interaction (i.e., *R*^2^ = 0.01). During the first intervention session, Lola demonstrated a small increase in sensitivity toward the availability of social interaction (i.e., *a* = 0.12). However, she also displayed more bias toward the side on which the therapist did not begin the session (i.e., *b* = −0.56) and her time allocation did not vary any more systematically with the availability of social interaction (i.e., *R*^2^ = 0.01).

Arthur’s data are displayed in the top row of the right group of graphs. During his last baseline session, he allocated his time indifferently (i.e., *a* = 0) and displayed extreme bias toward the side on which the therapist began the session (i.e., *b* = 2). Arthur’s time allocation still varied relatively systematically with the availability of social interaction (i.e., *R*^2^ = 0.6). During the first intervention session, Arthur demonstrated an increased sensitivity toward the availability of social interaction (i.e., *a* = 0.75) and bias toward the side on which the therapist did not begin the session (i.e., *b* = −1.03). However, his time allocation varied slightly less systematically with the availability of social interaction (i.e., *R*^2^ = 0.47).

Axel’s data are displayed in the second row of the right group of graphs. During his last baseline session, he allocated his time indifferently (i.e., *a* = 0.03) and displayed bias toward the side on which the therapist began the session (i.e., *b* = 0.9). Axel’s time allocation did not vary systematically with the availability of social interaction (i.e., *R*^2^ = 0). During the first intervention session, Axel demonstrated an increased sensitivity toward the availability of social interaction (i.e., *a* = 0.69) and his time allocation varied far more systematically with the availability of social interaction (i.e., *R*^2^ = 0.82). However, he also displayed bias toward the side on which the therapist did not begin the session (i.e., *b* = −0.35).

Candace’s data are displayed in the third row of the right group of graphs. During her last baseline session, she allocated her time slightly avoidantly (i.e., *a* = −0.25) and displayed extreme bias toward the side on which the therapist began the session (i.e., *b* = 1.62). Candace’s time allocation varied somewhat systematically with the availability of social interaction (i.e., *R*^2^ = 0.4). During the first intervention session, Candace allocated her time socially and demonstrated increased sensitivity to availability of social interaction (i.e., *a* = 0.45) and a substantial reduction in bias toward the side on which the therapist began the session (i.e., *b* = −0.01). However, her time allocation varied slightly less systematically with the availability of social interaction relative to baseline (i.e., *R*^2^ = 0.26).

Travis’ data are displayed in the bottom row of the right group of graphs. During his last baseline session, he allocated his time indifferently (i.e., *a* = 0.02) and displayed a very slight bias toward the side on which the therapist began the session (i.e., *b* = 0.16). Travis’ time allocation did not systematically vary with the availability of social interaction (i.e., *R*^2^ = 0). During the first intervention session, Travis demonstrated a small increase in sensitivity to the availability of social interaction (i.e., *a* = 0.13) and a slight bias toward the side on which the therapist did not begin the session (i.e., *b* = −0.08). However, his time allocation still did not vary systematically with the availability of social interaction (i.e., *R*^2^ = 0.1).

Across participants, the mean change in sensitivity to the availability of social interaction was 0.36, indicating that sensitivity increased from the last baseline session (*M* = 0.01) to the first intervention session (*M* = 0.37). The mean change in bias toward a given side of the assessment, considering absolute values of log bias, was −0.48, indicating that the degree of bias decreased from the last baseline session (*M* = 0.81) to the first intervention session (*M* = 0.33). The mean change in *R*^2^ was 0.27, indicating that time allocation varied more systematically with the availability of social interaction from the last baseline session (*M* = 0.16) to the first intervention session (*M* = 0.43).

### 3.3. Across Session Analysis

**Figure 3:**
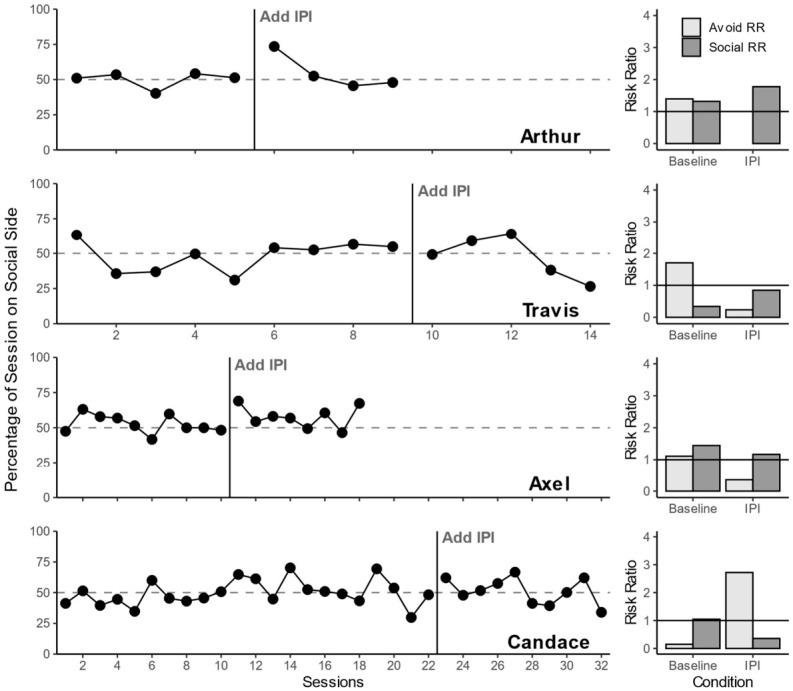
Percentage of Session on Social Side Across Sessions of Baseline and Intervention Phases with Corresponding Risk Ratios. Note: In the timeseries graphs (left), dashed horizontal lines correspond to 50% of the session spent on the social side, providing a reference point for indifferent time allocation. In the risk ratio graphs (right), solid horizontal lines correspond to a risk ratio of 1.0, reflecting no difference in the probability of switching sides following therapist movement relative to any other time.

Figure 3 displays the baseline and intervention data for Arthur, Travis, Axel, and Candace. Although the purpose of the current study was to use within-session matching analyses to evaluate intervention effects, Figure 3 facilitates direct comparison to the results presented by Morris and Vollmer (2022b). The left column displays the percentage of session on the social side, with phase lines indicating the onset of individualized preferred interaction. The right column displays the corresponding risk ratios for social and avoidant switches within each phase. Arthur’s data are shown in the top row. For Arthur, the introduction of individualized, preferred interaction produced an initial increase in allocation to the social side to approximately 70%; however, social time allocation decreased to baseline levels (approximately 50%) for the remainder of the intervention phase. We calculated non-overlap of all pairs (NAP; Parker & Vannest, 2009) to quantify effect size. Arthur’s time allocation data during intervention yield a value of 0.5. The likelihood of a social approach given therapist movement slightly increased, whereas avoidant switches became less likely during the intervention phase.

Travis’ data are shown in the second row. For Travis, there was no change in social time allocation across conditions, with allocation on the social side remaining near 50%. Toward the end of his intervention phase, a decreasing trend in the percentage of session on the social side was evident. Travis’ time allocation data during the intervention phase yield an NAP of 0.51. However, the risk ratio for avoidant switches decreased substantially from approximately 2 to near zero, indicating that he was less likely to display social avoidance following therapist movement during the intervention phase.

Axel’s data are shown in the third row. For Axel, the introduction of individualized, preferred interaction produced an initial increase in allocation to the social side to about 70%, but he rarely allocated more than 55% off session to the social side in subsequent sessions. Axel’s time allocation data during intervention yield an NAP of 0.67. During interventions sessions, the probability of social avoidance following therapist movement decreased relative to baseline. However, the probability of social approach following therapist movement also decreased relative to baseline.

Candace’s data are shown in the bottom row. Candace sometimes allocated a clear majority of the session to the social side during intervention sessions, but these high points did not exceed the range of data obtained during her relatively variable baseline. Candace’s time allocation data during intervention yield an NAP of 0.54. During intervention sessions, the probability of social approach following therapist movement decreased, while the probability of social avoidance following therapist movement increased relative to baseline (from an avoidance risk ratio near zero to approximately 2.7).

## 4. Discussion

### 4.1. Quantifying Intervention Effects Using the Generalized Matching Equation

The current study is a preliminary exploration of how the generalized matching equation may be used to quantitatively describe intervention effects. Our purpose was to apply within-session matching analyses to quantify the effects of individualized, preferred interactions on the social time allocation of children with developmental disabilities. Four out of seven participants displayed substantial increases in sensitivity to the availability of social interaction during the first intervention session relative to the last session of baseline. These increases were quantified by changes in *a* and were often accompanied by reductions in the degree of bias toward one side of the assessment (i.e., *b* closer to zero) and increases in the degree to which social time allocation systematically varied with the availability of social interaction (i.e., higher *R*^2^).

The present findings extend prior applications of the generalized matching equation to sociability assessments by demonstrating their utility for quantifying intervention effects. Previous research has applied matching analyses to quantify sensitivity to social reinforcement across multi-session conditions (Morris & Vollmer, 2022a) and based on the results of a single session (Morris & Pizzuto, 2023). However, these applications have been limited to characterizing baseline performance prior to any targeted intervention. The current study extends this approach by applying matching analyses to quantify changes in sensitivity, bias, and *R*^2^ following the introduction of individualized, preferred interactions. Although prior research has demonstrated that matching analyses can be used to quantify and understand the mechanisms of intervention effects (Kronfli et al., 2021), the current study extends the literature by quantifying within-participant changes in the parameters of the generalized matching equation to more precisely characterize whether and how intervention altered the relation between social interaction and time allocation.

Beyond demonstrating the feasibility of this analytic approach, the present findings highlight several potential advantages of applying matching analyses to evaluate intervention effects. First, matching analyses allow for quantitative characterization of intervention-related changes in behavior, which may facilitate comparison across participants and intervention strategies. For example, within-participant differences in *a*, *b*, and *R*^2^ obtained in the current study could provide a precise point of comparison for future research replicating this intervention or evaluating alternative intervention strategies such as mand training (e.g., Seaver & Bourret, 2020) or conditioning procedures to increase the reinforcing or discriminative value of social interactions (e.g., Jimenez-Gomez et al., 2024; Sainsbury et al., 2024; P. M. Taylor et al., 2025).

Second, the parameters of the generalized matching equation may help characterize the processes associated with changes in social time allocation. For example, increases in social time allocation may reflect enhanced sensitivity to social reinforcement (e.g., Axel), reductions in pre-existing bias toward one side of the arrangement (e.g., Candace), or more systematic allocation of behavior as a function of the availability of social play (e.g., Felicity). Changes in these parameters may generate hypotheses about the variables contributing to intervention efficacy or effectiveness, and may guide subsequent research designed to elucidate the mechanisms responsible for an intervention’s success or lack thereof. In the present study, the introduction of individualized, preferred interactions was most often associated with increased sensitivity to social reinforcement during the first intervention session. However, modifications to the baseline or intervention procedures may produce different patterns. For example, a more extended analysis in which the relative amount of the therapist spends on each side of the room is titrated across conditions (Morris & Vollmer, 2022a) may elucidate more systematic changes in bias and the degree to which time allocation systematically varies with social interaction.

Finally, the use of matching analyses may facilitate translation between basic and applied research by situating applied intervention effects within a well-established basic literature that has quantitatively characterized variables that influence choice. Interpreting intervention effects in terms of within-participant changes in sensitivity and bias connects applied data with basic experiments that may facilitate interpretation, analysis, and development of more efficacious intervention strategies. To illustrate, in the current study we did not include a changeover delay and delivered social interaction immediately following a switch to the social side. The basic literature suggests that this may have contributed to the pattern of frequent switching displayed (e.g., Felicity, Travis) given that, in the absence of a changeover delay, rapid alteration is frequently observed (e.g., Shull & Pliskoff, 1967; Temple et al., 1995). Future research should evaluate varied changeover delays within this experimental arrangement. Similarly, basic research suggests that incorporating ordinal discriminative stimuli (Bradshaw et al., 1976) and denser rates of reinforcement (Davison & Baum, 2000; Klapes et al., 2020) may improve sensitivity and the extent to which choice systematically varies with relative reinforcement rate. Future research should consider incorporating analogous procedures within this experimental arrangement by providing clear contingency-specifying rules prior to each session and increasing the rate at which individualized preferred interactions are initiated or offered.

### 4.2. Limitations and Potential Sources of Variability

The current study partially replicates findings from Morris and Vollmer (2022b), demonstrating that individualized, preferred interactions can increase social time allocation for three of the four participants recruited for the purpose of this study. However, unlike in Morris and Vollmer, these effects were only observed in the first session and did not maintain; social time allocation returned to baseline levels across subsequent sessions. This highlights three important limitations of the current study and our within-session matching analyses. First, especially given the inconsistent intervention effects observed, the current study is limited by a small sample size. Our sample size restricts the conclusions that can be drawn about the prevalence of different intervention outcomes and the utility of the within-session matching analyses in detecting them. The findings of the current study should be considered preliminary until these procedures have been replicated with a larger number of participants. Second, although a single session may be sufficient to draw conclusion about the effect of social interaction on time allocation, our findings illustrate that repeated measures are still necessary to evaluate the stability and durability of the effects obtained.

A third limitation is that the results of the within-session matching analyses did not systematically vary with social time allocation across repeated intervention sessions. In fact, some participants who displayed the largest improvements in sensitivity from the last baseline session to the first intervention session displayed the least maintenance in increased social time allocation across sessions (and vice versa). For example, the intervention produced meaningful, persistent improvements for Edward, Lola, and Felicity; however, the sensitivity parameters generated from their first session were lower than those observed for Axel, Arthur, and Candace, for whom the intervention effects were far less durable. This discrepancy suggests that early changes in sensitivity may not reliably predict sustained changes in social time allocation across sessions and highlights the importance of comprehensive, across session analyses. Future research could address the current study by extending the procedures of Morris and Vollmer (2022a) to include a generic interaction phase and individualized preferred interaction phase. It is possible that an across-session matching analysis may elucidate effects that could not be captured in the current study.

Variables that may account for heterogeneity in the magnitude and persistence of the effects of individualized preferred interactions are important to consider. One relevant variable may be the type of individualized preferred interactions. For some participants (e.g., Edward, Lola), two or more of their preferred interactions were highly physical interactions that may have restricted movement (e.g., begin picked up and spun around). For the remaining participants, only one or none of their preferred interactions were sufficiently physical as to restrict movement about the room. Although the therapist only initiated or offered preferred interactions to participants, the frequency with which movement-restrictive interactions are initiated could potentially help explain differences in intervention efficacy across participants.

Another variable that could help account for variability in intervention efficacy is the reinforcing efficacy of the individualized preferred interactions. In the current study, we only conducted preference assessments to identify the interactions to be included, which did not allow for clear conclusions about absolute or relative reinforcer efficacy of the preferred interactions. In future research, incorporating reinforcer assessments such as progressive ratio analyses (e.g., DeLeon et al., 2009; Morris et al., 2025; Roane, 2008) may help to quantify the reinforcing efficacy of individualized preferred interactions and account for variability in their efficacy as an intervention to increase social time allocation. However, previous research suggests that the highest preferred social interactions indicated by social preference assessments are often found to be the most efficacious reinforcers during subsequent reinforcer assessments (Morris et al., 2023). As a result, we evaluated whether the percentage of selections for the most highly preferred social interaction may be helpful in explaining heterogeneity in the magnitude and maintenance of increased in social time allocation, as measured by non-overlap of all pairs (Parker & Vannest, 2009).

**Figure 4:**
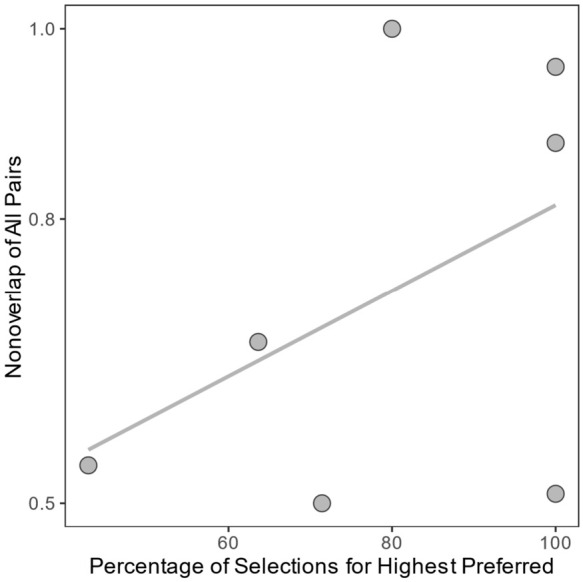
Relation between percentage of selections for the highest preferred social interaction and non-overlap of all pairs during the intervention phase.

Figure 4 displays the results of this analysis. The x-axis displays the percentage of selections for the most highly preferred social interaction, and the y-axis displays the non-overlap of all pairs effect size for individualized, preferred interactions. Each data point represents the values obtained for a single participant. These data suggest a positive correlation between the degree of preference for the highest preferred social interaction and the efficacy of highly preferred interactions (*r* = 0.45, 95% confidence interval: −0.45 to 0.89). The potential utility of this type of predictor variable can be characterized based on the sensitivity and specificity with which it predicts intervention outcomes (Falligant & Hagopian, 2020; Hagopian et al., 2018; Weber et al., 2024). All three participants for whom the intervention yielded an NAP > 0.8 selected their most-preferred social interaction at least 80% of trials during the preference assessment (i.e., sensitivity = 1). Whereas three out of four participants for whom the intervention yielded and NAP < 0.8 selected their most-preferred social interaction on less than 80% of trials during the preference assessment (i.e., specificity = 0.75). These analyses suggest that the percentage of selections for the most-preferred option could be a useful predictor of the efficacy of individualized preferred interactions for increasing time allocation. However, more data are needed to characterize the overall quality of this predictive relation and determine the exact percentage of selections that best delineates whether or not individualized preferred interactions will increase social time allocation.

### 4.3. Implications for Intervention Development

Ultimately, individualized preferred interactions may be useful as an initial, minimally intrusive intervention to increase social engagement. Our results illustrate that this intervention is likely to yield initial improvements in social time allocation but may often be insufficient to produce durable improvements that maintain across repeated sessions. When such transient effects are observed, this indicates that additional intervention components may be necessary to improve the reinforcing efficacy of social interaction. One approach may be to use conditioning procedures to directly alter the value of social interaction. For example, Axel and Travis, who participated in this study, went on to later participate in a Pavlovian conditioning intervention designed to improve the function of social interaction (P. M. Taylor et al., 2025). This subsequent intervention increased the relative reinforcer efficacy of social play for both participants. Thus, a similar progression of intervention components may be useful when individualized preferred interactions alone prove insufficient. Researchers have yet to use sociability assessments to evaluate the more general effects of Pavlovian conditioning procedures on the function of social interactions, which is an important direction for future research.

## 5. Conclusions

The current study brings together and builds upon previously disparate areas of research to demonstrate how within-participant changes in the parameters of the generalized matching equation can be used to quantify intervention effects. These analyses precisely characterized how individualized, preferred interactions altered participants’ time allocation based on changes in *a*, *b*, and *R*^2^ which, respectively, facilitated conclusions about the extent to which time allocation was sensitive to social interaction, biased toward a given side of the assessment arrangement, or influenced by variables beyond social interaction. Although individualized, preferred interactions often produced immediate improvements, these effects were not consistently maintained across sessions. Overall, the current study illustrates how quantitative analyses of behavior may complement traditional single-case timeseries analyses, help to understand heterogeneity in intervention efficacy, and facilitate the development and improvement of interventions for fundamental social behavior.

## Figures and Tables

**Table 1:** Participant Demographics and Primary Form of Communication.

Participant	Race/Ethnicity	Age (Years)	Sex	Diagnosis	Primary Form of Communication
Edward	-	7	Male	Autism Spectrum Disorder	Limited picture exchange
Felicity	-	5	Female	Autism Spectrum Disorder	Limited speech generating device
Lola	-	3	Female	Autism Spectrum Disorder	Limited picture exchange
Arthur	Black, non-Hispanic	5	Male	Autism Spectrum Disorder	Extensive vocal mands
Axel	White, non-Hispanic	3	Male	Developmental Delay, Hydrocephaly, and Aggressive Childhood Behavior	Limited gestures
Candace	Black, non-Hispanic	4	Female	Autism Spectrum Disorder	Extensive vocal mands
Travis	Black, non-Hispanic	4	Male	Autism Spectrum Disorder	Limited vocal mands and gestures

Note. Information regarding Edward, Felicity, and Lola’s race/ethnicity were not obtained by Morris and Vollmer (2022b).

## Data Availability

The original contributions presented in this study are included in the article. Further inquiries can be directed to the corresponding author.

## Notes

1 The independent play side of the assessment has historically been referred to as the alone side. We use the term independent play because it more accurately describes the conditions on that side of the room, where participants often engaged with toys or in stereotypic behavior despite the absence of social interaction.

2 Note that Morris and Vollmer (2022b) only included six options in their preferences assessment: five forms of social interaction and one control option.
